# Mindfulness and AI adoption: extending the technology acceptance model for Chinese media students

**DOI:** 10.3389/fpsyg.2025.1637502

**Published:** 2025-09-03

**Authors:** Yanling Lan, Sihang Liu, Hao Chen, Linjie Xia

**Affiliations:** School of Film Television and Communication, Xiamen University of Technology, Xiamen, China

**Keywords:** mindfulness, AI technology, media students, TAM, media education

## Abstract

**Introduction:**

Media students' acceptance and use of intelligent technology requires not only external environmental support but also the stimulation of internal driving forces. This study incorporates mindfulness into the classical Technology Acceptance Model (TAM) to investigate its role in shaping students' perceptions and behavioral intentions toward artificial intelligence (AI) tools. Specifically, the research examines: (1) the impact of mindfulness on media students' perceptions of AI technology; (2) the influence of mindfulness on their continuous intention to use AI technology; and (3) the moderating effect of perceived risk on the intention to adopt AI tools.

**Methods:**

Based on a conceptual model integrating mindfulness with TAM, this study conducted an offline questionnaire survey among 588 media students. The data were analyzed using SmartPLS and SPSS to test the structural equation modeling and moderating effects.

**Results:**

The findings revealed three key outcomes. First, mindfulness exerts a significant, direct, and positive influence on personal innovativeness (PI), perceived usefulness (PU), and perceived ease of use (PEU). Second, PI functions as a mediating variable in the relationship between mindfulness and AI-based behavioral intention (AIBI). Third, perceived risk (PR) significantly weakens the relationships between PI, PU, and AIBI.

**Discussion:**

This study demonstrates that mindfulness enhances media students' intention to adopt AI tools by strengthening their perceptions of usefulness, ease of use, and personal innovativeness. However, perceived risk undermines these positive effects. By integrating mindfulness into the Technology Acceptance Model (TAM), this research extends the theoretical understanding of AI technology acceptance and provides practical insights for media education. The findings highlight that embedding mindfulness training and reducing perceived risks can effectively foster rational acceptance and the innovative application of AI tools, thereby contributing to the cultivation of intelligent media talent.

## 1 Introduction

The rapid development of the “intelligent +” media ecosystem presents higher challenges and demands for media professionals. As the future talent for the industry, media students must actively embrace the wave of artificial intelligence, improve their digital literacy, and apply AI technologies to expand their professional abilities. Although the number of AI-related courses offered in media programs at Chinese universities has gradually increased, the effectiveness of these courses has been less than satisfactory. The primary reason for this is that most courses focus on the technology itself and are teacher-driven, neglecting the stimulation and cultivation of students' subjectivity and intrinsic motivation (Zhang, 2022b). Research focusing on media students' perception of AI technologies and their use of AI tools to assist learning, complete tasks, and engage in practical activities is limited (Tang et al., 2025). To effectively improve intelligent media education courses, and to enhance the cultivation of media talent in higher education, it is crucial to precisely segment student groups, consider individual characteristics, analyze students' cognition and behavior, and explore the factors and mechanisms influencing their acceptance of AI technologies. These elements should serve as the foundation for improving educational outcomes in intelligent media programs.

In recent decades, mindfulness has become increasingly popular as an individual psychological trait that centrally reflects intentional awareness. A number of studies have shown that it has a significant effect on the willingness to accept and use emerging technologies (Sun et al., 2016). In the process of promoting the use of intelligent technology, mindfulness can also alleviate the pressure brought by technology to individuals and promote a positive perception and acceptance of technology, to further promote individual wellbeing through the use of technologies (Klussman et al., 2020). At the same time, mindfulness also has a significant impact on students' learning ability, professional quality, technology application, and other important areas in higher education (Hämäläinen et al., 2021). Integrating mindfulness into the analysis of college students' intention to use artificial intelligence can both expand the dimension of students' technology acceptance models (Fareed and Kirkil, 2025) and improve the understanding of how technology is perceived. We can also confirm the internal factors that can stimulate students' positive perception and adoption of intelligent technology.

It is both necessary and urgent to understand how mindfulness influences the adoption and practical use of AI technology by media students in the digital age. However, there is a significant lag in introducing AI literature and teaching AI technology within the media education curriculum in Chinese universities. Specifically, there is a lack of empirical research exploring the factors that affect students' intention to use AI from the perspective of individual psychological traits (Song, 2024; Zhu et al., 2024). Therefore, this study integrates mindfulness into the classical Technology Acceptance Model (TAM) and analyzes its moderating effect using partial least squares structural equation modeling (PLS-SEM), based on the data collected from the questionnaire survey on Chinese media students. This study investigates the impact of mindfulness on Chinese media students' perceived usefulness, perceived ease of use, personal innovation, and behavioral intention of AI, as well as the moderating role of perceived risk. By focusing on the psychological trait of mindfulness, this study offers new insights into enhancing AI adoption among media students and provides curriculum design strategies and psychological intervention recommendations for cultivating intelligent media talent in the future.

## 2 Literature review

### 2.1 AI Technology in the media education

By reshaping media organizational structures, operational practices, and core tasks, AI-based media production is becoming increasingly prevalent. This transformation in how users interact with and consume media will bring disruptive and challenging influences to the future media industry (Carlson, 2015). The technical complexity and diversity of media production in the AI era highlight the urgency of reforming traditional media education (Zhang, 2022a). Baptista et al. (2025) argue that the rapidly developing “intelligent +” media industry imposes new requirements on media professionals, who now need to possess interdisciplinary knowledge, expertise in intelligent technologies, data analysis skills, and the ability to create content quickly. Pavlik (2023) advocates for the establishment of interactive media education systems based on AI technologies to guide students in effectively collaborating with AI.

However, in current media education, the integration of artificial intelligence still tends to focus primarily on technological development, neglecting the cultivation of students' individual intelligent thinking and professional confidence (Li et al., 2022). As a result, educational methods lag behind the evolving needs of the intelligent media industry (Hu et al., 2018). Furthermore, the failure to foster students' intrinsic motivation and proactive engagement in the acceptance of intelligent technologies has led to suboptimal teaching outcomes (Zhang, 2022b). Liu (2024) argues that, in the context of media education, it is crucial to accurately assess students' individual characteristics, analyze their behaviors, and address their personal awareness and attitudes. Niu et al. (2024) suggest that exploring the factors influencing students' willingness to adopt intelligent technologies will allow for the development of targeted strategies. Research by Tahat et al. (2023) analyzed the willingness of media students to use digital technology based on the Technology Acceptance Model (TAM), highlighting that perceived usefulness and perceived ease of use remain critical factors affecting ICT acceptance. Additionally, Soliman et al. (2025) emphasize the importance of considering students' personal attitudes toward the use of generative AI (GenAI) tools and creating interactive learning experiences to help students become well-informed citizens in an AI-driven world.

### 2.2 Mindfulness in the acceptance of AI technology

Teo et al. (2011) were the first to suggest that high levels of mindfulness promote organizational adoption of technology and enhance the efficiency of its application. Poston and Kettinger (2014) argue that mindfulness not only influences how new technologies are introduced, but also improves user satisfaction and the ability to understand technology. Sun et al. (2016) assert that a user's state of mindfulness is a critical factor in determining whether technology can be used consistently after initial exposure. Pflügner and Maier (2020) found that employees with higher levels of mindfulness experienced lower levels of technology-related stress, including overload, complexity, intrusiveness, and uncertainty. A recent study by Jang et al. (2021) found that mindfulness has a direct positive impact on both technology use and job performance in technology-intensive environments. For college students, mindfulness is a key factor in understanding their willingness to accept technology (Corti and Gelati, 2020). Mindfulness enhances students' creative learning and innovative acceptance, which, in turn, increases their willingness to use technology (Yeh et al., 2019).

With the development and widespread adoption of artificial intelligence, research on the specific impact of an individual's mindfulness level on the acceptance of intelligent technology has gradually emerged (Chuang et al., 2024). Hurst (2021) points out that providing users with personalized mindfulness experiences can effectively enhance digital skills in applications and tools; thus, digital mindfulness has been proposed as a mental health intervention and applied with increasing urgency. Rohwer et al. (2022) argue that technological stress is becoming more prevalent in the digitization of work, and both mindfulness and mindfulness in technology use positively affect the reduction of technological stress. Gupta et al. (2023) advocate for the development of mindfulness-based digital competency frameworks to support the sustainability of digital capabilities. Chen et al. (2024) confirmed the effect of mindfulness on emotional orientation and the intention to continue using Chat-GPT both during and after its application.

### 2.3 TAM in the adoption of AI technology

The Technology Acceptance Model (TAM), first proposed by Davis in 1989, is a theory of rational behavior, self-efficacy, and expectation confirmation, designed to explain how individuals adopt and use new technologies, particularly in the workplace. Davis et al. extended TAM by incorporating behavioral intentions as a component, which enhanced its explanatory power regarding user behavior. In subsequent research, Venkatesh further extended the TAM framework, developing the TAM2 model in 2000 and the Unified Theory of Technology Acceptance and Use (UTAUT) in 2003. By now, TAM remains one of the foundational theories for studying individuals' intentions and behaviors related to technology acceptance and usage, as it provides a detailed explanation of decision-making processes in technology adoption.

Recent studies have shown that the theoretical frameworks of research on college students' use or rejection of AI technology are primarily based on extensions of the classical TAM (Roy et al., 2022; Chen et al., 2024; Bekturova et al., 2025). Soliman et al. (2023) analyze the factors influencing the use of metaverse learning platforms by integrating TAM and Self-Determination Theory (SDT), suggesting that these factors significantly affect the adoption of metaverse platforms. Dahri et al. (2025) examine college students' willingness to use AI technology by combining SDT and TAM. Ma (2025) integrates personal innovativeness with TAM to explore the key factors affecting Chinese postgraduate students' willingness to use Chat-GPT, confirming that perceived usefulness (PU), perceived ease of use (PEU), and personal innovativeness (PI) have positive effects. Zhang et al. (2025) integrate TAM with Expectation-Confirmation Theory (ECT) and the Theory of Reasoned Action (TRA) into a comprehensive model, finding that performance expectation (PE) and technological innovation readiness index (TRII) have a significant positive impact on postgraduates' willingness to continue using AI. Al-Zahrani and Alasmari (2025) investigate middle school students' willingness to use AI in universities across the Middle East and North Africa region. Guided by TAM and the Diffusion of Innovations (DoI) theory, they find that PU, PEU, and personalized learning are significantly correlated with students' intention.

### 2.4 Research gaps

Based on research in media education, mindfulness, and the acceptance of AI, it has been confirmed that mindfulness plays a crucial role in the process of individuals' adoption of intelligent technology. However, most studies have focused on identifying the elements of mindfulness that affect technology acceptance, and there is a lack of a systematic theoretical framework and model construction. The investigation of mindfulness within the Technology Acceptance Model (TAM) is still in an exploratory phase. These researches are not only limited in number but also relatively simplistic in approach, often focusing on a one-dimensional, linear mechanism through which mindfulness influences the intention to use technology. Furthermore, studies examining how mindfulness impacts the college student's adoption of intelligent technology, particularly the students majoring in media, are still with a notable absence of systematic empirical studies.

Given the lack of a comprehensive framework across the three subjects including media education, mindfulness, and AI technology adoption, this study aims to explore whether and how the level of mindfulness affects media students' intention to accept and use intelligent technology, utilizing the mindfulness scale and the classical TAM. First, the role and function of mindfulness will be considered. Second, the internal structural mechanisms of mindfulness in relation to technology adoption will be examined. The main research questions are as follows:

RQ1: Does mindfulness affect media students' intention to adopt AI technology?

RQ2: If so, what are the factors and mechanisms by which mindfulness affects media students' intention? Are there other influencing factors?

In answering these questions, this paper focuses on how to enhance the internal drive of media students to use technological skills through the training and cultivation of mindfulness.

## 3 Theoretical framework and research hypothesis

### 3.1 Technology acceptance model (TAM)

The Technology Acceptance Model (TAM) includes several key variables, such as perceived usefulness (PU), perceived ease of use (PEU), behavioral intention (BI), and other external factors (Venkatesh and Davis, 1996). Among these, PU and PEU are the core variables, effectively explaining users' perceptions of new technology. PU refers to the user's belief that using the technology can improve performance, while PEU refers to the user's perception of how easy the technology is to use (Kim et al., 2009; Bischof-dos-Santos et al., 2017). Given the robustness of TAM, subsequent studies have largely extended the model, combining various research subjects and contexts to explain users' acceptance of new technologies (Bouaguel and Alsulimani, 2022). Recently, scholars have added personal innovativeness (PI) and perceived risk (PR) to TAM to better explain students' acceptance of AI technologies (Yu et al., 2024b; Liu et al., 2024). Lee et al. (2021) confirmed that PI is positively associated with students' intention to use AI, while Pan et al. (2024) explored the role of PR, finding that it moderates the relationship between PU and PEU The integration of factors such as PI and PR not only extends the classical TAM model but also provides more targeted insights into the adoption of artificial intelligence (Almeida et al., 2025).

### 3.2 Mindfulness

Mindfulness plays a key role in addressing negative emotions, cognitive challenges, and improving cognitive flexibility (Vogus and Sutcliffe, 2012; Yang et al., 2024). Moreover, mindfulness increasingly influences the analysis of psychological characteristics such as individuals' mindsets, values, beliefs, and emotions Wamsler and Brink, (2018). Recent studies have integrated mindfulness into the analysis of individuals' perception, application, and evaluation of new technologies (e.g., Oeldorf-Hirsch and Chen, 2022). Through this integration with the Technology Acceptance Model (TAM), it has been shown that mindfulness significantly impacts perceived usefulness, ease of use, and actual willingness to use technology. Specifically, it influences the cognitive processes of understanding, perceiving, and implementing AI technology, improving users' perceptions of its feasibility and accessibility. By strengthening the psychological influence, mindfulness promotes the usability, ease of use perception, and actual use behavior of AI technology (Tan, 2025).

### 3.3 Hypotheses development and research model

**Figure 1:**
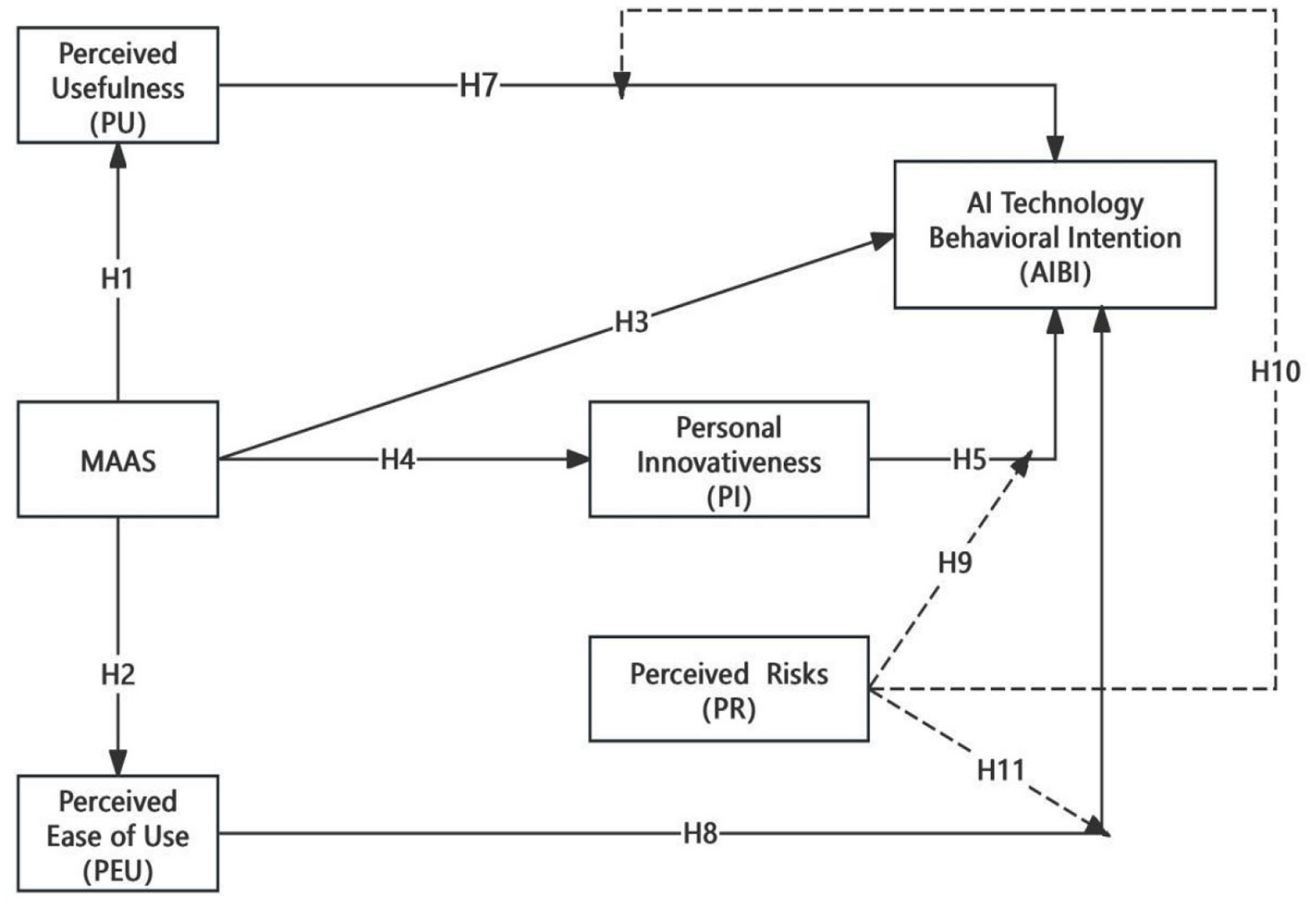
Structural equation modeling framework based on TAM and MS.

This study systematically examines the impact of mindfulness levels on media students' intention to use artificial intelligence (AI) technology by integrating mindfulness into the classical Technology Acceptance Model (TAM). It explores the relationship between mindfulness levels and the intention to use AI technology, proposing that mindfulness significantly influences perceived usefulness (PU), perceived ease of use (PEU), and the actual intention to use technology. Additionally, by incorporating personal innovativeness (PI) and perceived risk (PR) into TAM, the study identifies other important factors that predict students' intention to adopt AI technology. Figure 1 illustrates the theoretical model constructed in this study.

#### 3.3.1 Mindfulness and TAM of AI technology

Students' use of AI technology in educational settings is both challenging and risky (Teo et al., 2011). (Wamsler and Brink 2018) demonstrate that in higher education, students with higher levels of mindfulness are more likely to enhance their PU and PEU of new technology, ultimately increasing their actual willingness to use it (Jang et al., 2021). Recently, Long et al. (2025) found that students who engage in mindfulness training can overcome negative emotions, gain a deeper self-understanding, and improve both PU and PEU. Based on these findings, this study proposes the following research hypothesis:

Hypothesis 1: Mindfulness positively influences media students' perceived usefulness of AI technology.

Hypothesis 2: Mindfulness positively influences media students' perceived ease of use of AI technology.

Hypothesis 3: Mindfulness positively influences media students' behavioral intention to adopt AI technology.

#### 3.3.2 Personal innovativeness

Individual innovativeness refers to the ability to generate and promote creative ideas, implement innovations, and embrace new technologies in the work environment (Janssen, 2005). Students' intention to innovate with technology is influenced by intrinsic personal characteristics (Bernárdez et al., 2023), and enhancing personal innovativeness through mindfulness can be effective (Zhang et al., 2023). Recent research by Güğerçin and Kuss (2025) indicates that mindfulness has a significant positive impact on users' personal innovativeness. Building on these findings, this study proposes the following research hypothesis:

Hypothesis 4: Mindfulness positively influences media students' personal innovativeness in AI technology.

Earlier research on technology acceptance has linked personal innovativeness (PI) to actual willingness to use technology (Thompson et al., 2006; Lu et al., 2008). Students who exhibit higher levels of personal innovativeness tend to have a more positive attitude toward smart technology and a stronger willingness to use it (Chae et al., 2023). Çelik and Ayaz (2024) integrated TAM with PI to explore college students' intention to use the metaverse in higher education. Liu and Hu (2025) examined the factors influencing college students' use of online learning technologies and found that PI positively impacts students' continuance intention. Based on this body of research and the latest findings, the following research hypothesis is proposed:

Hypothesis 5: Personal innovativeness positively influences media students' behavioral intention to use AI technology.

Based on Hypotheses 3, 4, and 5, it can be inferred that personal innovativeness mediates the relationship between mindfulness and the actual intention to use technology. A recent study by Wang and Wu (2025) found that mindfulness influences digitalization behavior by enhancing personal innovation, further confirming the mediating role of individual innovativeness between mindfulness and the intention to use technology. Furthermore, Khari and Bali (2024) and Li et al. (2023) similarly demonstrate that individual innovativeness mediates the relationship between mindfulness and actual behavioral intention to use. Building on these findings, this study proposes the following research hypothesis:

Hypothesis 6: Personal innovativeness mediates the relationship between mindfulness and media students' behavioral intention to use AI technology.

#### 3.3.3 Perceived usefulness and perceived ease of use in TAM

PU and PEU are core variables in the TAM. First, when users perceive the usefulness of AI technology, their satisfaction and wellbeing are significantly enhanced, which ultimately increases their intention to use the technology (Mouakket, 2015). Algerafi et al. (2023) found that perceived usefulness positively influences the intention to continuously use intelligent assistive tools in a study of college students. Second, perceived ease of use is positively correlated with the intention to use AI technology (Bilquise et al., 2023). Zhao et al. (2024) concluded that perceived ease of use significantly and positively impacts students' willingness to use technology, based on an analysis of students' specific learning task scenarios. Building on the above research, this study proposes the following research hypothesis:

Hypothesis 7: Perceived usefulness has a positive and significant impact on media students' behavioral intention to use AI technology.

Hypothesis 8: Perceived ease of use has a positive and significant impact on media students' behavioral intention to use AI technology.

#### 3.3.4 The moderating effect of perceived risk

Perceived risk, first proposed by Bauer (1960), refers to the risk individuals anticipate regarding the outcome of an action before it is taken. Shin et al. (2017) and Zhang et al. (2021) highlight the importance of considering the impact of perceived risk in AI education. Perceived risk shifts individuals' focus from the innovative potential of technology to anxiety and concerns, ultimately diminishing the effect of personal innovativeness on the intention to use technology (Li et al., 2024). Jo (2024) found that perceived risk influences users' personal innovativeness and negatively impacts their purchase intention for Chat-GPT. Moreover, Bitton et al. (2024) identified a significant, negative moderating effect of perceived risk on the relationship between personal innovativeness and use intention. Based on these findings, this study proposes the following research hypothesis:

Hypothesis 9: Perceived risk negatively moderates the effect of personal innovativeness on media students' behavioral intention to use AI technology.

The current study indicates that negative emotions and future-oriented fear are significantly heightened when users perceive risks associated with the use of artificial intelligence technologies (Hong, 2025). These negative emotional responses, such as anxiety and fear, reduce users' PU and PEUof AI technologies, ultimately diminishing their actual intention to use these technologies (Yu et al., 2024a). Wu et al. (2022), using a sample of Chinese students, also reveal that perceived risk significantly negatively moderates the relationship between students' perceived usefulness of technology and their actual willingness to use it. Gieselmann et al. (2025) assert that when individuals perceive a higher risk associated with technology, the relationship between perceived ease of use and actual intention to use is significantly weakened. Based on these findings, this study proposes the following research hypothesis:

Hypothesis 10: Perceived risk negatively moderates the impact of perceived usefulness on media students' behavioral intention to use AI technology.

Hypothesis 11: Perceived risk negatively moderates the effect of perceived ease of use on media students' behavioral intention to use AI technology.

## 4 Materials and methods

### 4.1 Population and sample

The participants in this study were Chinese media students, including students from the majors in journalism and communication, television and film, and digital media art at both the undergraduate and graduate levels. Compared with the use of AI in majors like engineering and medicine, the adoption of AI technologies such as multimodal content generation, automated editing, and content creation has introduced a new generation of the media industry. These advancements bring about fundamental changes and even disruptive transformations (Nguyen and Hekman, 2022). Furthermore, when media students use artificial intelligence technologies, they must heavily rely on their perceptions and evaluations of the technology's usefulness, controllability, and value to ensure the ethical integrity and content accuracy of the generated material (Newman, 2022). As a result, media students face higher psychological complexity and situational sensitivity compared to students from other majors when using AI tools.

The minimum sample size for this study was determined using the G^*^Power tool (Faul et al., 2009). With parameters set to a moderate effect size of 0.15, an error rate of 0.05, an effect strength of 0.8, and 7 predictors, the minimum sample size required for credible results was 103. The sample was selected using stratified random sampling, with strata based primarily on participants' major and academic year, ensuring a representative distribution of students across the study's relevant subgroups.

This study utilized the Questionnaire Star platform to design and distribute the questionnaire in March 2025. The distribution was conducted offline, with face-to-face administration, on-site completion, and collection. Of the 610 returned questionnaires, invalid responses were identified and excluded based on the following criteria: (1) key items were missing or unanswered, and (2) the response time was significantly shorter than the average time (i.e., < 1 min). After excluding 21 invalid responses, 588 valid questionnaires remained, yielding a response rate of 96.6%. For data analysis, SmartPLS (V4.1) was used to assess the direct and indirect effects among variables in the structural equation model, while the Process macro in SPSS 26 was applied for moderation analysis.

### 4.2 Instruments

The questionnaire consists of two sections: the first section gathers basic demographic information from participants, and the second section assesses the variables in the model, including mindfulness, perceived usefulness, perceived ease of use, personal innovativeness, perceived risk, willingness to use, and actual use. A Likert scale was used to measure these variables. The specific test items are as follows:

#### 4.2.1 Mindfulness scale

The Mindful Attention Awareness Scale (MAAS), developed by Brown and Ryan (2003), was used to assess individuals' mindfulness characteristics. The scale consists of 15 items, each rated on a six-point scale. Chen et al. (2021) adapted the MAAS by using a 5-point Likert scale. The original MAAS employed an even-numbered scoring system, which could result in neutral respondents selecting inaccurate options. This issue is effectively addressed by the 5-point Likert scale (Gu et al., 2022). Consistent with Chen et al. (2021), this study utilizes the 5-point Likert scale to measure students' mindfulness, with 1 representing a lower mindfulness level and 5 representing a higher mindfulness level. The Cronbach's alpha for the mindfulness scale in this study was 0.921.

#### 4.2.2 Perceived usefulness scale

The Perceived Usefulness (PU) scale was developed by Davis (1989), and its reliability was reported to be 0.98, demonstrating high convergent, discriminant, and factorial validity. Wang and Shin (2022) introduced perceived usefulness into the analysis of usage intention for metaverse education platforms, designing a 5-point Likert scale ranging from “Strongly Disagree” to “Strongly Agree” with four items. In this study, the Cronbach's α value for the Perceived Usefulness scale was 0.908 (see Table 1).

**Table 1:** Analysis of reliability and validity about questionnaire.

**Variables**	**Items**	**FL**	**Cronbach's alpha**		**KMO**
**MAAS**	MA1	0.929	0.93		0.949
	MA2	0.926			
	MA3	0.924			
	MA4	0.925			
	MA5	0.925			
	MA6	0.927			
	MA7	0.921			
	MA8	0.921			
	MA9	0.922			
	MA10	0.921			
	MA11	0.927			
	MA12	0.924			
	MA13	0.929			
	MA14	0.923			
	MA15	0.927			
**Perceived usefulness**	PU1	0.926	0.898		
	PU2	0.899			
	PU3	0.885			
**Perceived ease of use**	PEU1	0.885	0.865		
	PEU2	0.899			
	PEU3	0.897			
**Personal innovativeness**	PI1	0.955	0.718		
	PI2	0.767			
	PI3	0.729			
**Perceived risks**	PR1	0.837	0.808	Corrected chi square	14,137.272
	PR2	0.851			
	PR3	0.844			
**AI technology behavioral intention**	AIBI1	0.897	0.857	*P*	< 0.001
	AIBI2	0.901			
	AIBI3	0.847			

#### 4.2.3 Perceived ease of use scale

The Perceived Ease of Use (PEOU) scale was also developed by Davis (2009), and its reliability was reported to be 0.904. Huang and Teo (2020) introduced perceived ease of use into the analysis of Chinese teachers' intention to use technology in education, designing a 5-point Likert scale ranging from “Strongly Disagree” to “Strongly Agree” with four items. In this study, the Cronbach's α value for the Perceived Ease of Use scale was 0.858 (see Table 1).

#### 4.2.4 Personal innovativeness scale

Initially created by Agarwal and Prasad (1998), this scale measures an individual's acceptance of new technology. Castiblanco Jimenez et al. (2021) revised the scale and proposed a 5-point Likert scale with three items, ranging from “Strongly Disagree” to “Strongly Agree.” In this study, the Personal Innovativeness scale has a Cronbach's α value of 0.858 (see Table 1), with the overall reliability being 0.702.

#### 4.2.5 Perceived risk scale

Seo and Lee (2021) further explored the relationship between perceived risk and AI technology, revising the influence of awareness and perceived risk on the Technology Acceptance Model (TAM). They developed a 5-point Likert scale with three items, ranging from “Strongly Disagree” to “Strongly Agree.” In this study, the Perceived Risk Scale has a Cronbach's α value of 0.799.

#### 4.2.6 Actual usage intention scale

First proposed by Trafimow and Finlay (1996), this scale measures an individual's intention to actually use technology. Wang and Chen (2024) adapted this scale to explore trust and acceptance of artificial intelligence (AI) technology, incorporating AI technology features. The revised scale is a 5-point Likert scale with three items, ranging from “Strongly Disagree” to “Strongly Agree.” In this study, the Actual Usage Intention Scale has a Cronbach's α value of 0.85.

## 5 Data analysis

In this study, SmartPLS (V4.1) software and partial least squares (PLS) were used to evaluate the theoretical model. PLS-SEM is widely employed across various fields, including education, economics, and computer science, for assessing large and complex models (Hair et al., 2021). PLS-SEM follows a causal prediction paradigm, which aligns with the objective of testing the predictive power of well-developed theoretical models (Sarstedt et al., 2022). It has strong predictive capabilities, with composite model properties that allow for both path estimation based on sample data and further predictive analysis through the computation of latent variable composites (Hair et al., 2019). Compared to other structural equation models, PLS-SEM offers greater accuracy in predictive power and stronger statistical performance, making it particularly suited for analyzing the impact of emerging technological factors (Donath et al., 2024).

Additionally, the Process macro (Momel 1) in SPSS 29 software (Hayes and Scharkow, 2013) was used, in conjunction with PLS-SEM, to examine whether perceived risk moderates the relationships between perceived usefulness, perceived ease of use, individual innovativeness, and actual intention to use. The Process macro utilizes ordinary least squares regression and bootstrapping to automatically construct interaction terms once the model framework is specified. The robustness of parameter estimates is assessed through bootstrap sampling, set to 5,000 iterations in this study. By generating bias-corrected confidence intervals, the reliability of the estimated results is enhanced (Chen et al., 2023). It also helps identify specific numerical intervals where the moderating effects are significant and outputs interaction slope diagrams, which visually illustrate the direction and strength of the moderation effect (Hayes, 2017). The simplicity and robustness of the PROCESS macro make it an ideal tool for analyzing moderating effects in studies on students' actual intention to use artificial intelligence (Wu et al., 2024; Toros et al., 2024).

To further address potential common method bias (CMV) in this study, we employed two approaches. First, we used the variance inflation factor (VIF) to assess multicollinearity. All VIF values for the internal model were below the threshold of 3.3, indicating that CMV is not a concern in this model (Kock, 2015). Second, following the approach of Rönkkö and Ylitalo (2011), we compared the results of the PLS marker model with the baseline model. The *R*^2^ changes were minimal: PU = 1.5%, AIBI = 5.4%, and PEU = 2%. Overall, the *R*^2^ changes were below the 10% threshold, suggesting that CMB does not significantly affect the model. These two approaches demonstrate that CMB is not a significant issue in this study.

### 5.1 Demographic description analysis

The gender distribution of the survey sample is as follows (see Table 2): 22% male, with a total of 130 males, and 78% female, with a total of 458 females. Regarding the educational level, 84% of the participants are undergraduates, with a total of 493 students. In terms of majors, the distribution is as follows: 241 students majoring in Drama and Film, 159 students in Digital Media Arts, and 188 students in Journalism and Communication.

**Table 2:** Frequency analysis of demographic variables.

**Variable**	**Option**	**Percentage**	**Mean**	**Variance**
Gender	Man	22.00%	1.78	0.173
	Women	78.00%		
Qualification	Undergraduate	84.00%	1.16	0.145
	Postgraduate	15.00%		
Major	Drama, film and television	41%	8.90	0.809
	Digital media	27%		
	Journalism and communication	32%		

### 5.2 Model reliability and validity testing

To assess model reliability, composite reliability (CR) and average variance extracted (AVE) were calculated. Table 3 shows CR values ranging from 0.838 to 0.935, all exceeding the recommended threshold of 0.70 (Urbach and Ahlemann, 2010). Most AVE values were above the recommended level of 0.50, with the AVE for mindfulness slightly below the threshold at 0.488. However, it still met the criteria when considered alongside its CR value of 0.934.

**Table 3:** Reliability and convergence effectiveness of variables.

**Reliability**		**Convergent validity**	
**Variables**	**Cronbach alpha**	**CR**	**AVE**
AIBI	0.853	0.911	0.773
MA	0.927	0.933	0.484
PEU	0.858	0.913	0.778
PI	0.71	0.838	0.634
PU	0.908	0.935	0.784

To further assess the convergent validity of the structural equation model, this study conducted a supplementary analysis using the square root of the AVE values. The results (see Table 4) show that the square root of the AVE values for all first-order variables are higher than the correlation coefficients between each variable and the other variables. This further indicates that discriminant validity is good.

**Table 4:** Fournier lackel standard.

**Fournier-Lackel values**	**AIBI**	**MA**	**PEU**	**PI**	**PU**
**AIBI**	0.879				
**MA**	0.198	0.698			
**PEU**	0.618	0.118	0.882		
**PI**	0.654	0.211	0.578	0.796	
**PU**	0.725	0.138	0.701	0.552	0.908

An HTMT analysis of the scales was conducted using SmartPLS. The HTMT ratio values obtained (see Table 5) indicate that all the variables have HTMT values < 0.85, suggesting that the variables exhibit good discriminant validity statistically. This means that the variables are conceptually independent and can individually explain different aspects of the research. Therefore, from the perspective of discriminant validity, these variables are both valid and reliable for use in the study.

**Table 5:** HTMT ratio numerical.

**HTMT values**	**AIBI**	**MA**	**PEU**	**PI**	**PU**
**AIBI**					
**MA**	0.181				
**PEU**	0.719	0.103			
**PI**	0.838	0.217	0.740		
**PU**	0.828	0.118	0.798	0.691	

### 5.3 Multicollinearity test

The data collected through the survey may be subject to common method bias. To address this issue, a multicollinearity test was performed. The six factors extracted for this study were analyzed for collinearity. The results (see Table 6) indicate that all variables had VIF values lower than 3.3. According to Kock's perspective, a VIF >3.3 is considered indicative of pathological multicollinearity, which could be affected by common method bias and contamination. This finding suggests that the measurements in this study are not significantly affected by covariance issues.

**Table 6:** VIF of latent variables.

**VIF values**	**AIBI**	**MA**	**PEU**	**PI**	**PU**
**AIBI**					
**MA**			1.000	1.000	1.000
**PEU**	2.194				
**PI**	1.602				
**PU**	2.101				

### 5.4 Model acceptance and interpretability

Table 7 presents the results of the theoretical effect test for the model in this study. *R*^2^ is a measure of the explanatory power of the endogenous variables. An *R*^2^ >0.67 indicates strong explanatory power; an *R*^2^ between 0.33 and 0.67 indicates moderate explanatory power; and an *R*^2^ < 0.19 indicates weak explanatory power. Although the *R*^2^ values for the variables PI (Personal Innovativeness) and PEU (Perceived Ease of Use) are below 0.19, the model's SRMR (Standardized Root Mean Square Residual) is 0.058 (with SRMR < 0.8 indicating an acceptable model fit), and the NFI (Normed Fit Index) is 0.834 (with NFI closer to 1 indicating a better fit). Therefore, the structural equation model constructed in this study still possesses a certain level of explanatory power and persuasiveness.

**Table 7:** Theoretical effect size of *R*^2^.

***R*** ^**2**^		**F** ^**2**^				
**Theoretical effect sizes for R**^2^ **and F**^2^		**AIBI**	**MA**	**PEU**	**PI**	**PU**
**AIBI**	0.622					
**MA**				0.143	0.147	0.196
**PEU**	0.142	0.197				
**PI**	0.145	0.193				
**PU**	0.197	0.284				

### 5.5 Hypothesis testing

**Figure 2:**
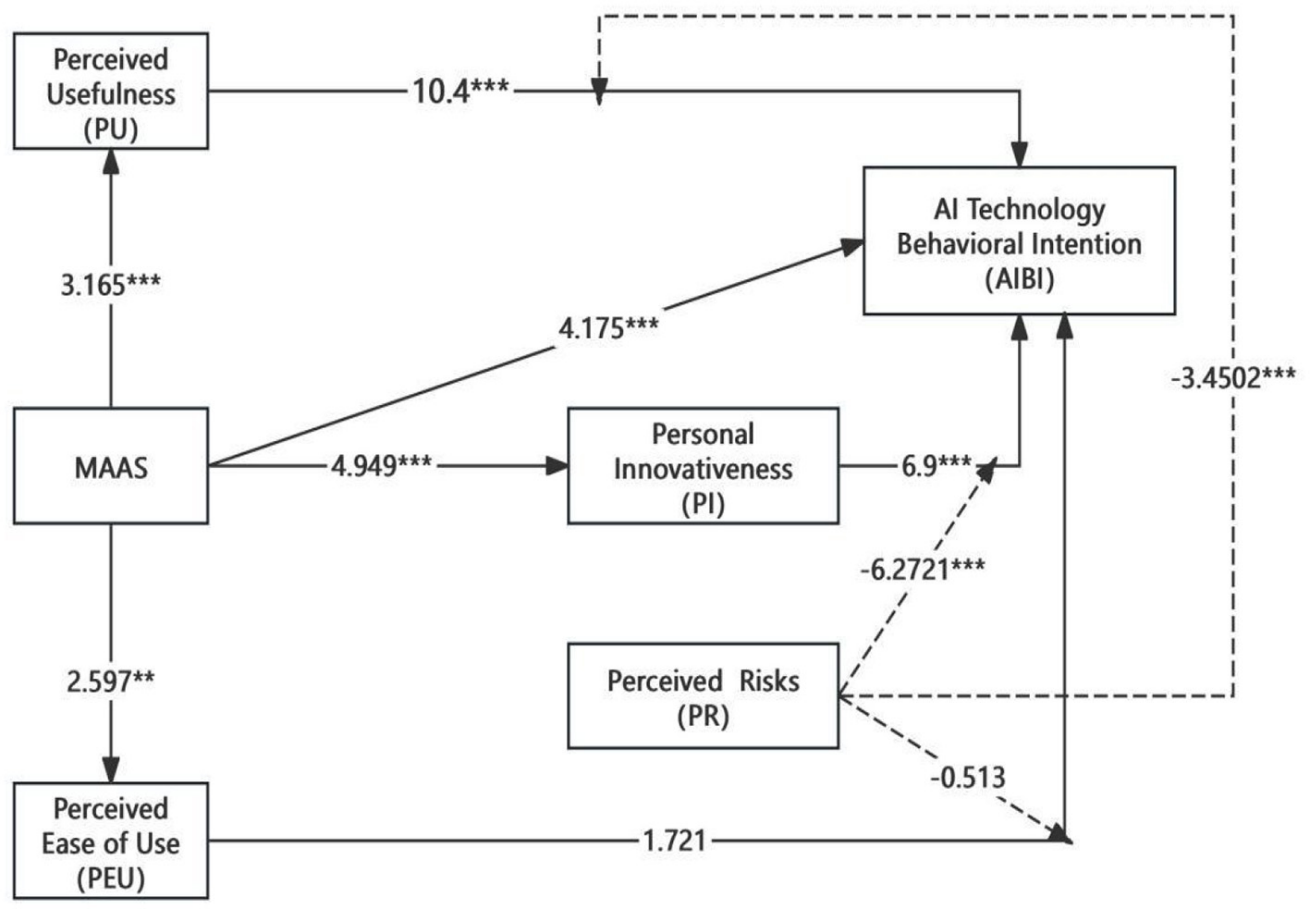
Structural equation path and significance. *p < 0.05; **p < 0.01; ***p < 0.001.

Using SmartPLS and SPSS29 software, the constructed model framework of this study underwent tests for direct effects, indirect path effects, total effects, and moderating effects. The final structural equation model, along with path coefficients and significance indices for each path, are shown in Figure 2.

Table 8 presents the variance, confidence intervals, *T*-values, and *P*-values (significance) for each path, calculated using the SmartPLS bootstrap method, with 5,000 re-samplings. From the data analysis, the relationships between mindfulness, perceived usefulness, perceived ease of use, personal innovativeness, and actual usage intention of artificial intelligence (AI) among media major students are clearly identified.

**Table 8:** Direct path effects table.

**Path coefficients of the research framework**	**Original Sample**	**Sample Mean**	**Confidence interval**		**STDEV**	**Significance**	
			**2.50%**	**97.50%**		***t***	***p***
**Direct effects**							
MA -> PEU	0.118	0.134	0.05	0.22%	0.046	2.597	0.009
MA -> PI	0.211	0.228	0.16	0.304	0.043	4.949	0
MA -> PU	0.138	0.154	0.08	0.232	0.043	3.165	0.002
PEU -> AIBI	0.088	0.087	0.013	0.186	0.051	1.721	0.085
PI -> AIBI	0.342	0.343	0.248	0.442	0.05	6.9	0
PU -> AIBI	0.475	0.474	0.38	0.563	0.046	10.4	0
**Indirect effect**							
MA-PU-AIBI	0.065	0.074	0.036	0.116	0.023	2.888	0.004
MA-PI-AIBI	0.072	0.078	0.047	0.116	0.019	3.785	0
MA-PEU-AIBI	0.01	0.012	0.002	0.03	0.008	1.233	0.218
**Total effect**							
MA-AIBI	0.148	0.164	0.109	0.227	0.036	4.175	0

Table 8 shows the variance, confidence interval, *t*-value, and *p*-value (significance) for each path, calculated using the SmartPLS bootstrap method and repeated 5,000 times. Therefore, through data analysis, the relationship between perceived usefulness, ease of use, personal innovation, and actual willingness to use artificial intelligence by college students majoring in mindful media can be clearly understood. Data analysis shows that the path coefficient of mindfulness on perceived usefulness is *p* = 0.002, *p* < 0.01 (SD = 0.043, *t* = 3.165), indicating that mindfulness has a significant, positive effect on perceived usefulness. In the impact of mindfulness on perceived ease of use, the path coefficient is *p* = 0.009, *p* < 0.01 (SD = 0.046, *t* = 2.597), indicating that mindfulness has a significant, positive effect on perceived ease of use. In the impact of mindfulness on the willingness to use artificial intelligence technology, the path coefficient is *p* = 0, *p* < 0.001 (SD = 0.036, *t* = 4.175), indicating that mindfulness has a significant, positive impact on the willingness to use artificial intelligence. In the impact of mindfulness on personal innovation, the path coefficient is *p* = 0.000, *p* < 0.001 (SD = 0.043, *t* = 4.949), indicating that mindfulness will have a significant, positive effect on personal innovation. In the impact of personal innovation on the actual use of artificial intelligence, the path coefficient is *p* = 0, *p* < 0.001 (SD = 0.046, *t* = 10.4), indicating that personal innovation has a significant, positive impact on the actual behavioral intention to use intelligent technology. In addition, the path coefficient *p* = 0, *p* < 0.001 (SD = 0.019, *t* = 3.785) in the process in which individual innovativeness exists as a mediator variable, and the path coefficient *p* = 0, *p* < 0.001 (SD = 0.019, *t* = 3.785), expresses that personal innovativeness does serve as an intermediate effect of mindfulness on the actual behavioral intention to use AI, and that mindfulness can moderate this effect by improving students' personal innovativeness, and successfully promote the acceptance and use of artificial intelligence tools. In the impact of perceived usefulness on the actual behavioral intention of artificial intelligence, the path coefficient *p* = 0, *p* < 0.001 (SD = 0.05, *t* = 6.9) indicates that perceived usefulness has a significant positive impact on the actual behavioral intention to use artificial intelligence. In the impact of perceived ease of use on the actual behavioral intention to use artificial intelligence, the path coefficient is *p* = 0.085, *p* > 0.05 (SD = 0.051, *t* = 1.721), indicating that perceived ease of use does not have a significant effect on the actual behavioral intention to use artificial intelligence.

Finally, the H1, H2, H3, H4, H5, H6, H7, H9, and H10 hypotheses proposed in this study are effectively supported, while H8 and H11 are not effectively verified.

**Figure 3:**
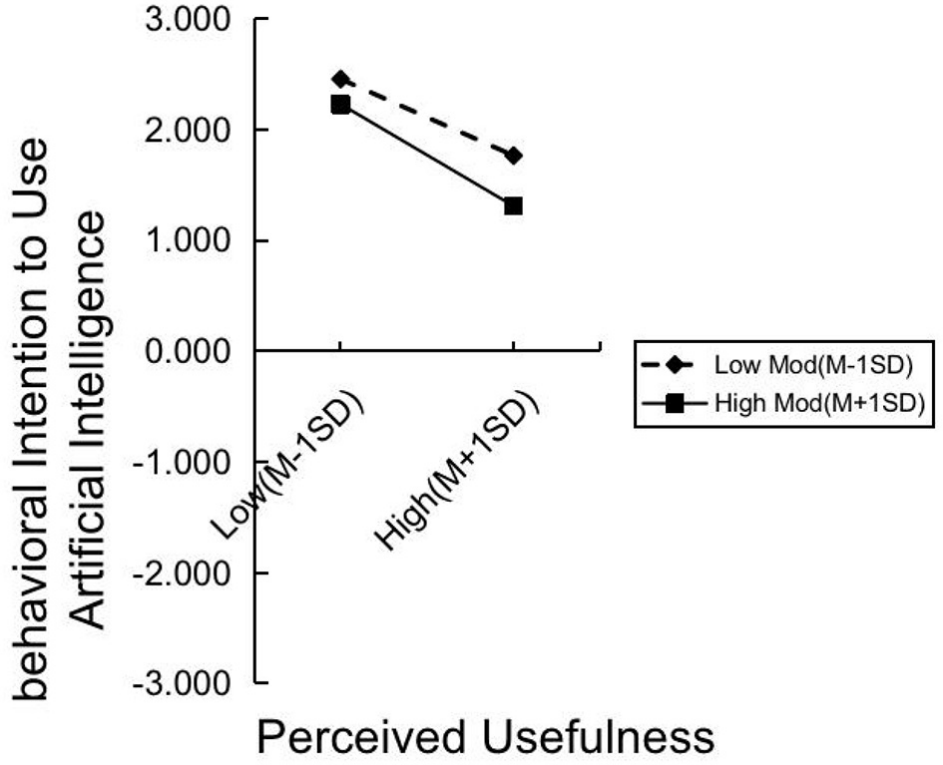
PRxPU AIBI simple slope plot.

**Figure 4:**
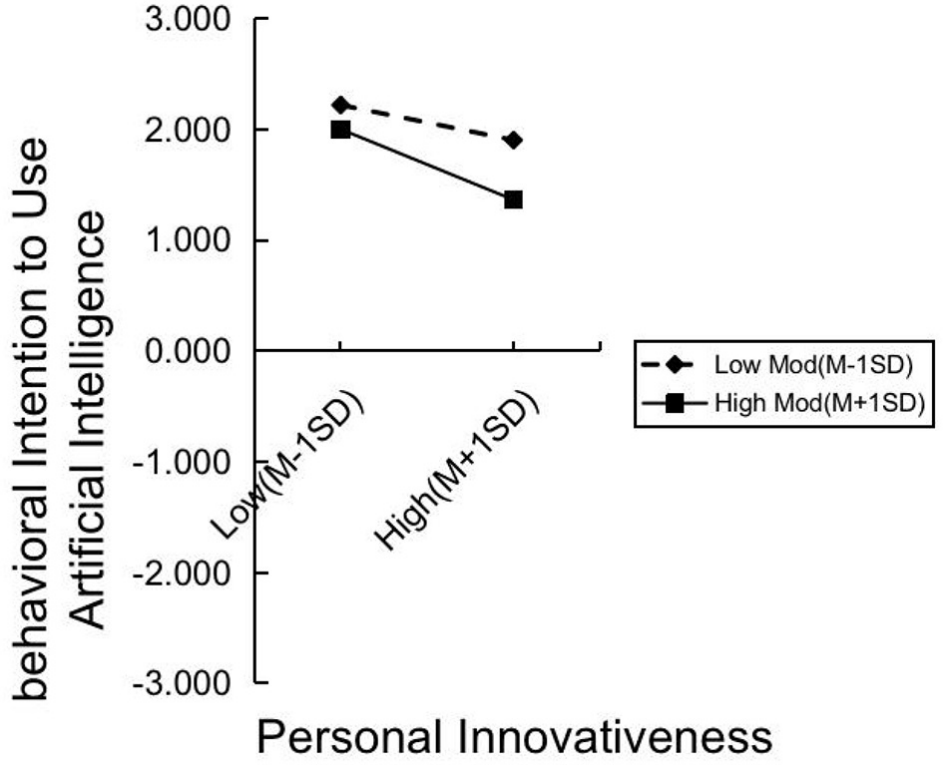
RRxPI-AIBI simple slope plot.

The Process macro in SPSS 29 was used to test the moderating effect, with the results presented in Table 9. Additionally, simple adjustment slope charts were created using Excel software, as shown in Figures 3, 4. The results reveal that perceived risk has a significant negative moderating effect on the relationship between perceived usefulness and actual intention to use (*t* = −3.4502, *p* = 0.0006, < 0.001). Perceived risk also significantly moderates the relationship between personal innovativeness and actual use intention (*t* = −6.2721, *p* = 0.000, < 0.001). However, perceived risk does not significantly moderate the relationship between perceived ease of use and actual usage intention (*t* = −0.513, *p* = 0.9591).

**Table 9:** Regulating effect pathway diagram.

**Moderating effect**	***t***	***p***	**LLCI**	**ULCL**	***R***	***R* ^2^**	**F**
RRxPU-AIBI	−3.4502	0.0006	−0.0933	−0.0257	0.4911	0.2412	11.9038^***^
PRxPEU-AIBI	−0.513	0.9591	−0.335	0.0318	0.3985	0.1588	0.0026
PRxPI-AIBI	−6.2721	0	−0.1123	−0.0588	0.3915	0.1533	39.3397^***^

^*^p < 0.05;

^**^p < 0.01;

*** *p* < 0.001.

## 6 Discussion

### 6.1 Direct effect influence mechanism driven by mindfulness

The research indicates that mindfulness has a significant positive impact on the perceived usefulness (PU) of intelligent tools among media students. This supports Hypothesis 1, aligning with the findings of Liebherr et al. (2024) and Dai et al. (2022). Mindfulness, by promoting a focus on the present moment, enhances the ability to organize and analyze problems clearly. It also facilitates the secretion of dopamine and promotes wellbeing, which buffers against external distractions (Marsh et al., 2024), thereby increasing individuals' perceived usefulness of technology. Similarly, Hypothesis 2 is also supported, showing that the higher the level of mindfulness, the stronger the perceived ease of use of AI tools. This finding is consistent with previous studies (Ameer and Zubair, 2020; Liu et al., 2025), which suggest a positive spiral between mindfulness and emotions. As mindfulness levels increase, individuals experience more positive emotions, which help them overcome technology-related anxiety. This, in turn, leads to a more active evaluation of the technology's utility and ultimately enhances the perceived ease of use. Finally, Hypothesis 3 is also effectively supported. The findings indicate that mindfulness plays a crucial role in enhancing students' actual intention to use technology, a conclusion that resonates with studies by Galante et al. (2016) and Hämäläinen et al. (2021).

This study supports Hypothesis 4, providing empirical evidence for a significant, positive relationship between mindfulness and personal innovativeness. This aligns with the findings of Jobbehdar Nourafkan et al. (2022) suggest that individuals with higher levels of mindfulness tend to exhibit greater creativity, which, in turn, drives students to engage more consciously in cultivating personal creative abilities. Additionally, Henriksen et al. (2020) assert that mindfulness training, regardless of duration, enhances personal innovativeness by fostering creativity. Furthermore, Hypothesis 5 of this study demonstrates a significant positive relationship between personal innovativeness and students' intention to use AI technologies. This finding is supported by previous research (Kasilingam, 2020; Sowmya et al., 2023), which shows that personal innovativeness, as an innate trait, drives students to adopt new technologies more readily. This aligns with Strzelecki et al. (2024), who suggest that individuals exhibiting higher personal innovativeness exhibit behaviors that reduce the anxiety and stress typically associated with new technologies like AI, thus promoting acceptance and enhancing the intention to use AI tools (Kwarteng et al., 2023).

This study assumes effective support for H6. This suggests that mindfulness not only directly influences actual intention but also facilitates the adoption of new technologies by enhancing personal innovativeness. High levels of mindfulness improve cognitive abilities, such as memory and attention, which foster innovative thinking and maintain innovation-driven behaviors, leading to a greater willingness to adopt and use new technologies during learning tasks (Golubickis et al., 2024). Conversely, individuals with low levels of mindfulness find it more challenging to recognize and adjust to changes in their emotional states. When in a negative emotional state, their personal innovativeness is diminished, which ultimately decreases their willingness to use AI technologies (Petrou and Jongerling, 2022).

### 6.2 Perceived usefulness, perceived ease of use, and behavioral intention

The findings of this study not only reinforce the central role of perceived usefulness in predicting actual use intention, as established in previous TAM research, but also underscore its significance in shaping users' technology adoption behavior. Specifically, students' perception of the usefulness of artificial intelligence tools enhances their expectation confirmation, leading to the belief that these tools are effective in task processing (Niu and Mvondo, 2024), which in turn increases their willingness to use them (Bilquise et al., 2023). When students perceive AI tools as highly useful, concerns related to AI ethical risks, privacy issues, and negative evaluations are mitigated (Camilleri, 2024), which strengthens their expectancy and self-efficacy. By reducing emotional barriers such as anxiety or worry, the perceived effectiveness of the tools is enhanced, ultimately fostering a greater willingness to use artificial intelligence technologies (Acosta-Enriquez et al., 2024).

Students' perceived ease of use of artificial intelligence technology does not significantly impact their actual usage intention, and research hypothesis 8 has not been effectively supported. Similarly, Soliman et al. (2024) found that perceived ease of use was not significantly related to the actual intention to use it. Zhao et al. (2025) also suggest that students' intentions may be influenced by various factors, including technology compatibility, facilitating conditions, and trust, which are more directly associated with behavior. They argue that focusing solely on perceived ease of use does not sufficiently explain actual usage intention. Recent studies by Oved and Alt (2025) also indicate that perceived ease of use is not the sole influencing factor; other external contexts, such as cultural, social, and technological environments, play a critical role in shaping users' actual intention to use.

### 6.3 The negative moderating effect of perceived risk

This study hypothesizes that H9 is effectively supported, indicating that perceived risk negatively moderates the relationship between individual innovativeness and actual usage intention. Perceived risk is a crucial factor, primarily due to students' fear and anxiety regarding the perceived rationality and safety of AI technology, which creates uncertainty in their cognition and application of intelligent technology (Ye et al., 2024). This heightened perception of risk exacerbates students' negative emotions, such as panic, worry, and anxiety, before they engage with the technology. As a result, it increases the pressure associated with using AI technology and inadvertently weakens the relationship between personal innovativeness and the actual intention to use it (Bullock et al., 2025).

This study also supports hypothesis H10, which posits that perceived risk negatively moderates the relationship between perceived usefulness and actual intention to use. Perceived risk exacerbates the distrust of AI technology among Chinese college students majoring in Media (Khan and Khan, 2019; Chung and Kwon, 2025). This distrust stems from negative assumptions regarding the use of AI technologies, including potential fears about their accuracy and effectiveness in smart education contexts (Wu et al., 2023). Additionally, Duffett et al. (2024) found that the higher the perceived risk of technology, the lower the positive impact of individual innovativeness on the intention to use it.

However, hypothesis H11 is not supported in this study, indicating that perceived risk does not significantly moderate the relationship between perceived ease of use and actual usage intention. This result is consistent with the findings of Li et al. (2024), who similarly found no significant moderating effect of perceived risk in this relationship. One possible explanation is that perceived ease of use is largely influenced by users' emotional experiences, such as feelings of depression, joy, or frustration, which are shaped more by individual emotions than by perceived risk (Mohamad et al., 2021). Similar conclusions have been drawn by Lei et al. (2022), Tilibasa et al. (2023), and Goel et al. (2025), who assert that the relationship between perceived ease of use and actual usage intention is more complex than previously assumed. Moreover, the high level of technological familiarity within the sample may have introduced bias, potentially limiting the model's capacity to detect significant moderating effects.

## 7 Practical implications

Based on individuals' levels of mindfulness, students' actual intention to use AI technology is significantly influenced. Media education in universities can incorporate mindfulness into various courses, including literacy, technical creation, and creative perception. This approach offers a novel and effective path for improving teaching outcomes and enhancing students' openness to intelligent tools. For instance, universities should introduce specialized courses in intelligent media that integrate mindfulness. These courses could feature short-term, structured mindfulness training modules designed to cultivate students' attention regulation, emotional awareness, and cognitive flexibility, especially in intelligent media-related subjects. Additionally, mindfulness can be incorporated into courses focused on technology creation and content generation. Practical activities, such as a GAI and Mindfulness Creativity Workshop, a Mindfulness-Awareness AI Interactive Creation Course, and a Technical Mindfulness Module Training Camp, can foster students' innovative thinking and creative abilities.

Moreover, it is important to address perceived risk, which influences the entire process of media students' engagement with intelligent technology. Perceived risk can trigger anxiety, skepticism, and even resistance. Therefore, universities should consider offering Digital Health Cognition Courses grounded in mindfulness training to enhance students' positive perceptions of intelligent technologies. These courses can help reduce barriers and alleviate psychological burdens caused by uncertainty and fear of technology. Combining mindfulness with communication strategies, such as popular science lectures led by experts in mindfulness and artificial intelligence, can also help students develop rational thinking and foster positive attitudes toward technology, counteracting anxiety and resistance.

Finally, as AI intelligent production replaces traditional media workflows, media students may experience significant concern. To address this, universities should develop Media Mindfulness Training Courses aimed at enhancing students' autonomy, critical thinking, and active engagement with AI technologies. These courses can help students overcome passive or negative attitudes toward artificial intelligence, fostering a mindset geared toward exploration and collaboration. Additionally, courses like Mindfulness Stress Regulation in Media could equip students with the tools to assess AI's potential risks and impacts on the future of the media industry, encouraging more informed and proactive use of these technologies.

## 8 Conclusion

Mindfulness plays a crucial role in enhancing perceived usefulness, perceived ease of use, and personal innovativeness. These factors, in turn, contribute to improving individuals' intention to use artificial intelligence. Beyond its direct positive impact on the intention to use AI, mindfulness also influences this intention indirectly through personal innovativeness, which acts as a partial mediator. Furthermore, perceived risk significantly moderates the relationship between perceived usefulness, personal innovativeness, and the behavioral intention to use AI, with a negative effect. By integrating mindfulness into the classical Technology Acceptance Model (TAM), this study bridges external technological factors and internal personal characteristics, offering a broader perspective on AI adoption. In doing so, it not only extends the research field but also provides an innovative framework for understanding the application and advancement of AI education and technology. Because of its simplicity, convenience, and applicability, mindfulness-based approaches in AI education hold substantial practical value and offer broad relevance.

## 9 Limitation and future research directions

Due to practical constraints related to human resources, time limitations, and material resources, this study faces the following limitations: (1) Limitations of the Research Methodology. This study exclusively employs structural equation modeling for empirical analysis, lacking qualitative data from interviews or focus groups that could provide deeper insights into media students' experiences with intelligent tools. Additionally, the absence of a cross-sectional design and other research methods limits the exploration of the diverse factors influencing students' use of smart technologies. (2) Limitations of the Research Sample. The sample consists solely of media students from universities in Fujian Province, China, which limits the generalizability of the findings. Expanding the sample to include a wider range of students from different regions and disciplines would improve the representativeness of the study and make the results more universally applicable. (3) Moreover, the study focuses only on mindfulness as a key factor among personal characteristics, without considering other variables such as intelligence, literacy, demographics, and learning ability. This oversight may limit the comprehensive understanding of respondents' personal traits. (4) Due to constraints in research conditions, a longitudinal approach was not employed, meaning that changes in mindfulness over time—especially after students have engaged with and learned to use intelligent technologies—were not accounted for. The absence of longitudinal data may prevent the study from capturing the dynamic nature of mindfulness development.

In light of the limitations of this study, the following suggestions are proposed for future research: (1) Expanding the sample size: future studies should broaden the sample size by incorporating students from universities and graduate schools across different regions, thereby enhancing the credibility, universality, and representativeness of the findings. (2) Incorporating additional variables: it is recommended that future research consider the inclusion of “literacy” as a key influencing factor and measurement indicator. This would allow for a more comprehensive assessment of the characteristics of learners, offering a more holistic view of the variables affecting students' use of intelligent technologies. Utilizing a mixed research design: combining questionnaire surveys with controlled experiments, along with both longitudinal and cross-sectional studies, would strengthen the validity of the conclusions and make the results more robust and generalizable. This mixed-method approach would offer a clearer understanding of the evolution of students' use of intelligent tools over time. In-depth qualitative research: future studies could enrich their findings by integrating in-depth qualitative research, such as focus group discussions or interviews with media students utilizing AI tools. This would provide deeper insights into students' perceived motivations and experiences before and after using smart tools. By implementing these strategies, future research could provide a more well-rounded and thorough exploration of the factors influencing media students' use of artificial intelligence.

## Data Availability

The raw data supporting the conclusions of this article will be made available by the authors, without undue reservation.
